# Discrimination of individuals in a general population at high-risk for alcoholic and non-alcoholic fatty liver disease based on liver stiffness: a cross section study

**DOI:** 10.1186/1471-230X-11-70

**Published:** 2011-06-13

**Authors:** Masaru Baba, Ken Furuya, Hideaki Bandou, Kenji Kasai, Kuniaki Sadaoka

**Affiliations:** 1 Hokkaido Social Insurance Hospital, Center for Gastroenterology and Hepatology, Sapporo, Japan

## Abstract

**Background:**

Factors associated with liver stiffness (LS) are unknown and normal reference values for LS have not been established. Individuals at high risk for alcoholic (ALD) and non-alcoholic fatty (NAFLD) liver disease need to be non-invasively discriminated during routine health checks. Factors related to LS measured using a FibroScan and normal reference values for LS are presented in this report.

**Methods:**

We measured LS using a FibroScan in 416 consecutive individuals who presented for routine medical checks. We also investigated the relationship between LS and age, body mass index (BMI), liver function (LF), alcohol consumption, and fatty liver determined by ultrasonography. We identified individuals at high-risk for ALD and NAFLD as having a higher LS value than the normal upper limit detected in 171 healthy controls.

**Results:**

The LS value for all individuals was 4.7 +/- 1.5 kPa (mean +/- SD) and LS significantly and positively correlated with BMI and LF test results. The LS was significantly higher among individuals with, than without fatty liver. Liver stiffness in the 171 healthy controls was 4.3 +/- 0.81 kPa and the upper limit of LS in the normal controls was 5.9 kPa. We found that 60 (14.3%) of 416 study participants had abnormal LS. The proportion of individuals whose LS values exceeded the normal upper limit was over five-fold higher among those with, than without fatty liver accompanied by abnormal LF test results.

**Conclusions:**

Liver stiffness could be used to non-invasively monitor the progression of chronic liver diseases and to discriminate individuals at high risk for ALD and NAFLD during routine health assessments.

## Background

A large body of evidence supports the importance of liver biopsies for understanding the progression of chronic liver disease [1]. Although liver biopsy is the gold standard for diagnosing chronic liver disease and for determining the extent of liver fibrosis, adverse effects and sampling errors are associated with the procedure [2-7], because a standard liver biopsy samples only about 1/50,000 of the liver [8]. In addition, the role of the liver biopsy in nonalcoholic fatty liver disease (NAFLD) is controversial. Arguments against routine liver biopsy include the generally benign course of NAFLD, the absence of established effective therapies even when findings indicate a need for treatment, and the risks associated with liver biopsy [9].

To distinguish nonalcoholic steatohepatitis (NASH) from NAFLD in routine practice is difficult. Efforts have been made to diagnose NASH using various imaging studies and blood tests, but the outcome has remained inadequate [10].

The FibroScan (Echo Sens, Paris, France) is a new transient elastometer that can noninvasively measure LS [11,12]. Correlations between LS and fibrosis have been reported [13,14] and monitoring LS might provide a method for observing and judging the progression of chronic liver diseases, including fatty liver disease [15].

Various clinical factors other than fibrosis itself, including necroinflammation of the liver, cholestasis and congestive heart failure can affect LS regardless of the severity of fibrosis [13,14]. However, factors influencing LS in apparently healthy subjects other than liver fibrosis have not been sufficiently clarified [16-19]. Almost all European studies have focused on patients with chronic liver disease. Although a few studies have evaluated healthy individuals from the viewpoint of defining normal reference values for LS [16,17], some of those included might have had subclinical liver disease. Therefore, normal reference values for of LS still need to be established based on data from healthy persons who are considered not to have liver disease at any stage.

The present study explores factors related to LS, establishes normal reference values for LS in healthy individuals, and determines whether LS can help to identify individuals at high-risk for ALD and NAFLD.

## Methods

### Study population

This study enrolled 423 consecutive Japanese individuals between the ages of 20 and 68 years (250 males; hepatitis B surface antigen negative; hepatitis C virus antibody negative), who presented for annual medical checkups at our health examination centre between July 2004 and April 2006. We excluded individuals with a history of any liver or cardiac diseases (especially congestive heart failure) and those who were under treatment for such conditions. We also excluded those with platelet counts < 150,000/μL. A medical history was obtained from each of the 423 persons, who then underwent a physical examination, a chest X-ray, electrocardiography and standard laboratory tests including FBS and HbA1c. Seven were excluded because LS could not be measured due to extensive intercostal subcutaneous tissue depth. Finally, 416 individual (250 males) were enrolled in this study. All participants provided written informed consent to participate in this study, which proceeded according to the Declaration of Helsinki and under the approval of our institutional review board.

### Abdominal ultrasonography and liver stiffness measured by transient elastography

We used ultrasonography to diagnose fatty liver based on hepatorenal contrast visualization, increased ultrasonographic signals in the liver parenchyma and decreased signals from deep areas of the liver. We also confirmed the absence of space-occupying lesions in the liver. Stiffness was measured in the right lobe of the liver accessed using a FibroScan through the intercostal spaces of participants placed in the dorsal decubitus position with the right arm in maximal abduction. Technical and procedural details have been described [13,20]. In brief, the FibroScan is equipped with a probe that includes an ultrasonic transducer. The tip of the transducer is covered with gel and placed on a specific location. A low-frequency, mild amplitude vibration is transmitted to the liver from the tip of the transducer. This vibration induces an elastic shear wave, of which the propagation and velocity are measured by simultaneous ultrasonography and expressed in kilopascals (kPa). These properties are directly related to liver tissue stiffness. Higher values indicate increased liver stiffness. Up to 10 measurements were obtained from each participant, and LS is represented as median values. Only procedures with at least 10 valid measurements, a success rate of at least 60% and an IQR to median value ratio of < 30% were considered reliable [21]. The success rate and IQR in this study were 94% and 17%, respectively. The FibroScan measures LS in a volume that approximates a 4-cm long cylinder with a diameter of 1 cm located between 2.5 and 6.5 cm below the skin surface. This volume is about 100-fold larger than a standard liver biopsy specimen, and thus might be more representative of the entire hepatic parenchyma. Individuals (N = 7) with intercostal subcutaneous tissues ≥ 25 mm [11-13] were excluded from the study because liver stiffness could not be measured under such conditions using the FibroScan.

### Measurement of physical findings and liver function tests

Height and weight were measured and BMI was calculated. To further assess the impact of body weight, we assigned the 416 participants into groups based on BMI < 23, 23 to ≤ 25, and > 25. The thickness of the intercostal subcutaneous tissues was measured only to confirm whether LS could be measured. Daily amounts and duration of alcohol consumption were evaluated using a formal, written questionnaire and total amounts of consumed alcohol were calculated as quantities of pure alcohol. Since alcohol consumption of ≤ 20 g of per day is prerequisite for a definition of NASH, we assigned the 416 participants into groups based on daily alcohol consumption of ≤ 20 and > 20 g/day (non-/occasional and habitual consumption, respectively) [20,22]. Liver function (LF) was tested in terms of serum levels of aspartate 2-oxoglutarate aminotransferase (AST), alanine aminotransferase (ALT) and γ-glutamyltranspeptidase (γ-GTP). The normal upper limits of AST, ALT and γ-GTP at our health centre are 35, 35 and 55 U/L, respectively. We defined normal LF tests results as each of AST, ALT and γ-GTP being within normal limits.

### Influence of liver dysfunction and fatty liver on LS

The participants were separated based on LF tests results and the presence or absence of fatty liver into Group 1 (N = 260), normal LF and no fatty liver; Group 2 (N = 32), abnormal LF and no fatty liver; Group 3 (N = 60), normal LF and fatty liver; Group 4 (N = 59), abnormal LF and fatty liver.

### Relationship between LS and platelet counts or platelet ratio index

We counted platelets and calculated the AST to platelet ratio index (APRI) to determine the relationship between LS and apparent liver fibrosis in all participants [23].

### Determination of normal reference values for liver stiffness

**Figure 1:**
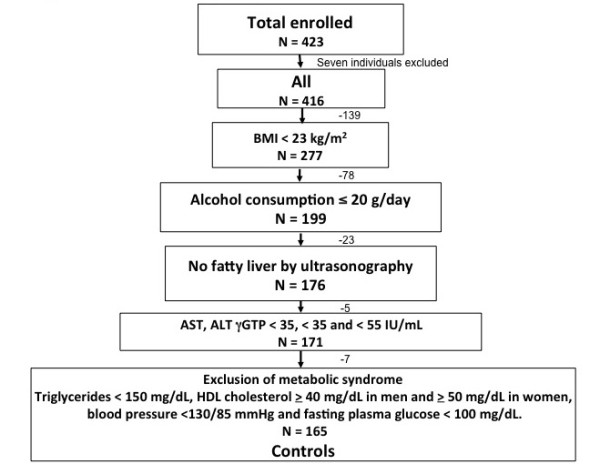
**Flow diagram of study protocol**. Seven individuals were excluded because LS could not be measured due to extensive intercostal subcutaneous tissue. We excluded 250 individuals based on four criteria and selected 165 healthy controls from the included population.

We selected healthy control candidates based on the following criteria: BMI < 23 kg/m^2^, normal LF, no findings of fatty liver by ultrasonography and daily alcohol consumption ≤ 20 g/day. We defined being overweight and obesity based on the World Health Organization (WHO) criteria for the Western Pacific Region as BMI ≥ 23 and ≥ 25 kg/m^2^, respectively [22,24]. None of our healthy control candidates fulfilled the criteria for metabolic syndrome proposed by International Diabetes Federation [25]. Furthermore, to establish normal reference values based on stricter criteria, candidates had to meet any one of the following conditions: triglycerides, < 150 mg/dL; HDL cholesterol, ≥ 40 mg/dL in men and ≥ 50 mg/dL in women; blood pressure, < 130/85 mmHg and fasting plasma glucose, < 100 mg/dL. We excluded six candidates because of high blood pressure. We finally selected 165 (77 males) of the 416 participants who met the criteria described above as normal controls (Figure 1)

### Statistical analysis

Continuous variables are expressed as means ± SD. The relationship between LS and each variable was assessed using Pearson's correlation coefficient and LS was compared between two groups using the unpaired Student's *t*-test. Differences among more than three groups were analyzed using the analysis of variance (ANOVA) when variables were normally distributed, followed by multiple comparisons. Categorical data were compared using the χ^2 ^test and the Cochran-Armitage test for trends. Both univariate and multivariate analyses were performed using logistic regression models. P < 0.05 was considered to represent a statistically significant difference. Data were statistically analysed using JMP 9.01 software (SAS Institute Inc., Cary, NC).

## Results

Table 1 shows the background characteristics of all participants and controls. The value for LS in all (N = 416) participants was 4.73 ± 1.54 kPa (range, 2.3-18.6 kPa) and that in healthy controls (N = 165) was 4.30 ± 0.81 kPa (range, 2.7-7.4 kPa) with a normal upper limit of 5.9 kPa (mean + 2SD). Liver stiffness tended to increase with age in all participants, although the difference did not reach statistical significance (p = 0.11).

**Table 1:** Characteristic of all subjects and controls.

	All subjects	Controls
Case(M/F)	416(250/168)	165(77/88)
Age(years)	47.4 ± 13.6	45.1 ± 14.4
BMI(kg/m^2^)	22.1 ± 3.1	20.2 ± 2.0
BW(kg)	60.1 ± 11.4	53.6 ± 8.3
Thickness of subcutaneous tissue(cm)	1.5 ± 0.4	1.2 ± 0.3
Alcohol consumption(g/day)	30.2 ± 44.3	6.3 ± 4.1
AST(U/L)	23.1 ± 10.9	20.4 ± 4.0
ALT(U/L)	23.5 ± 16.4	19.0 ± 6.6
γGTP(U/L)	40.6 ± 41.7	23.7 ± 10.5

Liver stiffness values were significantly higher in males than in females among all participants (4.88 ± 1.77 vs. 4.52 ± 1.11 kPa, respectively, p = 0.02), but did not significantly differ between healthy control males and females (4.41 ± 0.79 vs. 4.20 ± 0.83 kPa, respectively, p = 0.10).

**Figure 2:**
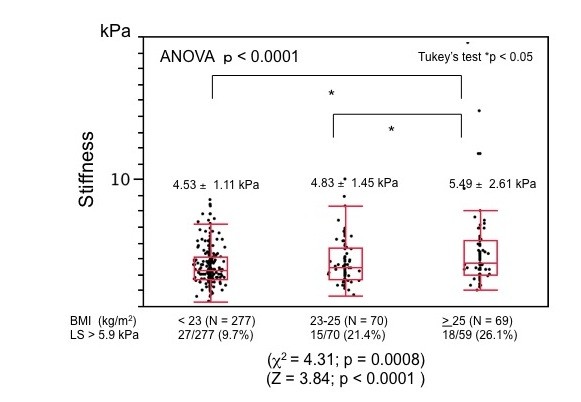
**Comparison of liver stiffness among three groups based on BMI**. Mean LS significantly differed among groups and were corrected for multiple comparisons according to Tukey's test. Proportions of individuals with LS above normal upper limit significantly differed (χ^2 ^test and Cochran-Armitage test for trends).

Liver stiffness significantly correlated with BMI in all participants (Table 2). Mean LS based on BMI (ANOVA p < 0.0001; Figure 2) and the proportion of those with LS beyond the normal upper limit (*x*^2 ^= 14.31, p = 0.0008) also significantly differed among the three groups. The Cochran-Armitage test for trend also showed a linear trend for proportions across these groups (Z = 3.84, p < 0.0001).

**Table 2:** The relationship between liver stiffness and other factors in all participants.

	Correlation coefficient(R)	P value
Age(years)	0.078	0.11
BMI(kg/m^2^)	0.258	<0.0001
BW(kg)	0.213	< 0.0001
Thickness of subcutaneous tissue(cm)	0.221	< 0.0001
Alcohol consumption(g/day)	0.011	0.07
Alcohol consumption(total)	0.023	0.64
AST(U/L)	0.231	< 0.0001
ALT(U/L)	0.346	< 0.0001
γGTP(U/L)	0.078	0.11
Plt(× 10^4^/μL)	0.001	0.38
APRI	0.11	< 0.0001

**Figure 3:**
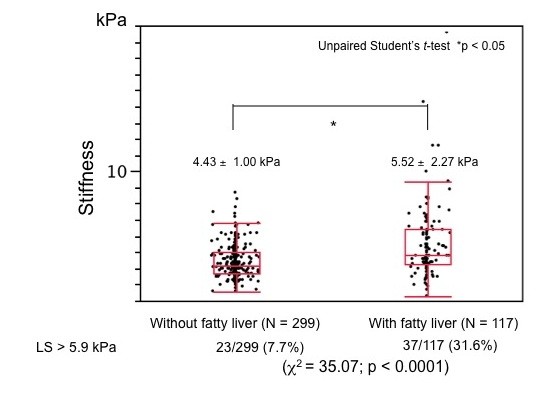
**Comparison of liver stiffness based on presence or absence of fatty liver**. Values for LS were significantly higher among individuals with, than without fatty liver. Ratios of those with LS above normal upper limit significantly differed (χ^2 ^test).

Values for LS were significantly higher among individuals with, than without fatty liver. Ratios of those with LS above normal upper limit significantly differed (*x*^2 ^= 37.54, p < 0.0001; Figure 3).

**Figure 4:**
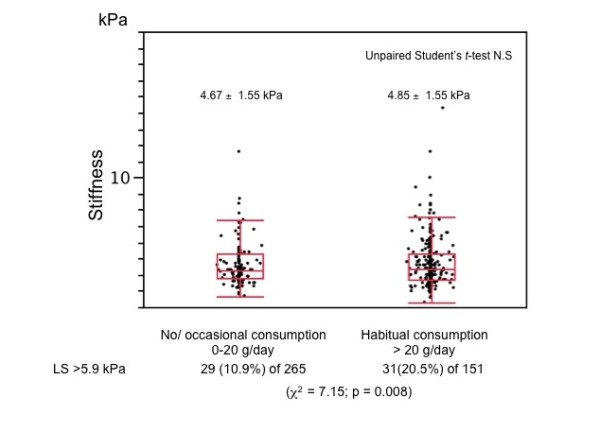
**Comparison of liver stiffness between two groups based on alcohol consumption**. Liver stiffness did not significantly differ between non/occasional and habitual consumption groups. However, ratios of those with higher LS than normal upper limit were significantly higher in habitual, than in non/occasional group (χ^2 ^test).

Liver stiffness did not correlate with daily or total alcohol consumption (Table 2) and did not significantly differ between those with no or occasional alcohol consumption, and those with habitual consumption. However the ratio of those with LS above the normal upper limit was significantly higher in the group who habitually consumed alcohol (*x*^2 ^= 7.15, p = 0.008; Figure 4).

Liver stiffness significantly and positively correlated with levels of AST or ALT, and with APRI but not with γ-GTP (Table 2) or platelet counts in all participants.

**Figure 5:**
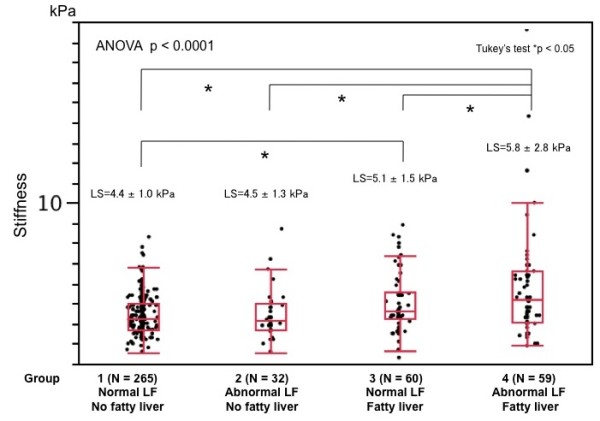
**Comparison of liver stiffness among four groups based on liver dysfunction and fatty liver**. Liver stiffness significantly differed among these groups. Liver stiffness was highest in Group 4 and that of Group 3 was higher than that of Group 1 according to multiple comparisons (Tukey's test).

**Figure 6:**
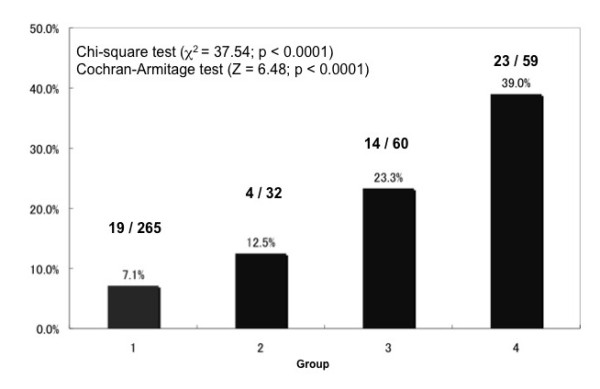
**Ratios of individuals in four groups with liver stiffness beyond normal upper limit**. Ratios of individuals with LS beyond the normal upper limit significantly differed (χ^2 ^test and Cochran-Armitage test for trend).

We found that 60 (14.3%) of the 416 participants had higher LS. We also found that LS significantly differed between the presence and absence of fatty liver (ANOVA, p < 0.0001) when the participants were assigned to four groups based on the results of LF tests. In addition, LS was the highest in Group 4, and higher in Group 3 than in Group 1 according to multiple comparisons (Figure 5). Nineteen (7.1%), 4 (12.5%), 14 (23.3%) and 23 (39.0%) participants in Groups 1, 2, 3 and 4, respectively, had LS values above the normal upper limit and the proportions significantly differed among the four groups (*x*^2 ^= 37.54, p < 0.0001). The Cochran-Armitage test for trend also showed a linear trend in proportions across the four groups (Z = 6.48, p < 0.0001; Figure 6).

**Figure 7:**
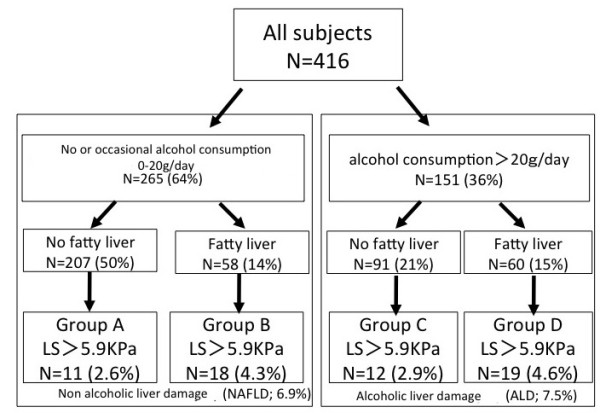
**Comparison of liver stiffness among four groups based on alcohol consumption and fatty liver**. All participants were assigned to two groups based on alcohol consumption and then each group was further divided into two subgroups with or without fatty liver according to ultrasonography (Groups A, B, C, D). About 7% (6.9% and 7.5%) of all participants had LS above 5.9 kPa among those with NAFLD or ALD regardless of fatty liver.

Figure 7 shows a comparison of liver stiffness among four groups based on alcohol consumption and fatty liver. All participants were assigned to two groups based on alcohol consumption and then each group was subdivided into two groups based on the presence or absence of fatty liver according to ultrasonography (Groups A, B, C and D). About 7% (6.9% and 7.5%) of all participants had LS above 5.9 kPa among those with NAFLD or ALD regardless of fatty liver. We could not exclude subclinical fatty changes that were undetectable by ultrasonography in Groups A and C. We thus speculated that liver stiffness can discriminate hepatic abnormalities more effectively than conventional ultrasonography.

Univariate analysis found that BMI, alcohol consumption, abnormal LF test results, fatty liver and APRI were associated with LS.

We excluded abnormal LF test results and fatty liver as confounding factors in multivariate analysis because these can result from a higher BMI or habitual alcohol consumption. We also excluded APRI because this merely reflected serum levels of AST in this study population. Finally, we included BMI and alcohol consumption in the multivariate analysis model.

Multivariate analysis revealed that BMI (23 - 25 kg/m^2^: odds ratio (OR), 2.21; 95% confidence interval (CI), 1.06-4.46; p = 0.033; ≥ 25 kg/m^2^: OR, 3.25; 95% CI, 1.62-6.43; p = 0.001) was the only independent predictor of higher LS (Table 3).

**Table 3:** Results of univariate and multivariate logistic regression analyses of factors affecting LS.

Univariate	Odds ratio	95% Cl	P value
Sex	1		
Female			
Male	1.56	0.93-2.62	0.08
Age(years)			
< 40	1		
40-60	1.5	0.82-2.77	0.17
≥60	1.54	0.71-3.29	0.27
BMI(kg/m^2^)			
< 23	1		
23-25	2.52	1.23-5.01	0.012
> 25	3.26	1.65-6.34	0.0008
Alcohol(g/day)			
0-20	1		
> 20	2.12	1.23-3.70	0.0075
Abnormal LFT	3.88	2.17-6.94	< 0.0001
Fatty liver	5.55	3.11-9.88	< 0.0001
APRI	2.11	1.06-3.23	0.0001

Multivariate	Odds ratio	95% CI	P value

BMI 23-25 kg/m^2^	2.21	1.06-4.46	0.033
BMI > 25 kg/m^2^	3.25	1.62-6.43	0.001
Alcohol (g/day)	0.97	0.50-1.82	0.94

## Discussion

We described factors related to LS and defined normal reference values for liver stiffness among healthy control individuals. We also showed that individuals at high risk for ALD and NAFLD could be discriminated by measuring LS using a FibroScan, which is useful for assessing patients with confirmed chronic liver disease [26]. However, few studies have examined its relevance to a first-line diagnosis of liver disorders and to identifying patients at high risk during routine medical checks.

The present study found no significant statistical differences between healthy control males and females, although all enrolled participants significantly differed. Whether gender should be taken into account when interpreting liver stiffness has been disputed, because several confounders between gender and BMI, metabolic syndrome, and alcohol consumption have not been appropriately adjusted [16,17,19].

We investigated which factors are related to LS before determining the normal upper limit of LS. Factors associated with LS in all participants were BMI, the presence of fatty liver, alcohol consumption and serum levels of AST and ALT. These findings suggested that being overweight (including obesity), habitual alcohol consumption and abnormal LF test results are important factors associated with a higher LS. We then selected those who met our criteria for normal controls based on these findings.

One of the major reasons for the higher LS in individuals with a high BMI seemed to be liver steatosis caused by NAFLD or ALD. Mean liver stiffness was higher in individuals with, than without fatty liver, but the distribution of LS widely overlapped between the two groups. We speculated that factors in addition to steatosis would be involved in increased LS. We considered that the mechanisms involved in the increase in the LS in our study population might be related to inflammatory cell invasion, liver cell degeneration and liver fibrosis as well as steatosis, all of which have been pathologically demonstrated in patients with NASH and ALD [27]. Whether or not simple steatosis alone would increase LS remains to be resolved.

The normal upper limit of LS was determined based on patients with a BMI < 23 kg/m^2 ^in this study. However, if the upper limit was based on the WHO global criteria for obesity of BMI ≥ 25 kg/m^2^, [28], then LS and the upper limit of LS in controls would be 4.44 ± 1.05 and 6.5 kPa, respectively.

Daily or total alcohol consumption significantly and positively correlated with γ-GTP levels in our study population (data not shown) as described [29,30]. The absence of correlations between LS and daily or total alcohol consumption can be explained by individual genetic differences in alcohol metabolism and the presence of a higher LS in patients with NAFLD [23,31]. The ratio of individuals with LS beyond the normal upper limit was significantly higher among those who habitually consumed alcohol than in those who did not consume, or who occasionally consumed alcohol. Therefore, alcohol consumption seems to be an important factor that affects LS. Although a normal upper limit of LS was established for participants who consumed ≤ 20 g of alcohol/day, LS and the upper limits of LS in controls (N = 91 of 165) who did not consume alcohol were 4.15 ± 0.66 and 5.5 kPa, respectively. This normal upper limit of LS was essentially equivalent to that reported by Kim et al. We considered that to determine the effect of consuming a very small amount of alcohol on LS in this population would be almost impossible, so we adopted alcohol consumption criteria of ≤ 20 g/day.

The present study found that LS was related to AST and ALT levels or APRI, which significantly correlates with stage of fibrosis and with a higher correlation coefficient than platelet count or AST levels alone in patients with chronic hepatitis C infection [32]. Because our study population was limited to individuals who presented for annual medical checkups, LS was more closely correlated with AST than with APRI, and did not associate with platelet counts. Liver damage that causes AST/ALT elevation also appeared to influence LS, because LS is higher even in patients with acute viral hepatitis but without liver fibrosis [33].

Univariate analysis indicated that BMI, alcohol consumption, LF test results and fatty liver status were associated with LS. Among these, the three BMI categories were significantly associated with fatty liver (χ^2 ^= 100.69, p < 0.0001). Alcohol consumption also significantly associated with abnormal LF test results (χ^2 ^= 26.8, p < 0.0001). We assumed that being overweight and habitual alcohol consumption can cause abnormal LF tests results and fatty liver, so BMI and alcohol consumption were included in the multivariate logistic regression model. Finally, BMI was identified, as the only independent predictor of higher LS and LS was significantly higher even in the group with BMI 23 - 25 kg/m^2^. Although interpreting LS might be subject to ethnic differences, we considered that our BMI criteria were appropriate for inclusion among normal reference values. Cobbold et al. have highlighted some issues regarding LS and BMI [19].

Table 4 summarizes two published reports and our study. Roulot et al. [16] examined a large cohort of apparently healthy individuals with no evidence of liver disease and established normal liver stiffness values (5.60 ± 1.30 kPa in males, 5.05 ± 1.49 kPa in females. Although their normal upper limit of liver stiffness were relatively high and almost corresponded to METAVIRE fibrosis stage 2 (LS = 7.2 kPa) [14,19].

**Table 4:** Comparison of three studies.

	**Roulet et al.**[16]		**Kim et al.**[17]		This study	
	**All subjects**	**Controls**	**All subjects**	**Controls**	**All subjects**	**Controls**

Number of subjects	429 (196 males)	370 (167 males)	73	69 (35 males)	416 (250 males)	165 (77 males)
Age	45.1 ± 16.7	43.3 ± 16.5	ND	38.9 ± 11.9	47.4 ± 13.6	45.1 ± 14.4
BMI	25.6 ± 4.2	24.9 ± 3.8	ND	22.6 ± 2.5	22.1 ± 3.1	20.2 ± 2.0

Population	Subjects for free medical checkups		Living doners for liver and kidney		Subjects for annual medical checkups	

Factors related to liver stiffness						

Age	NS (p = 0.06)	ND	ND	NS	NS (p = 0.11)	NS
Gender	p = 0.0002	p = 0.0006	ND	NS	p = 0.02	NS (p = 0.10)
BMI	p = 0.0005	ND	ND	NS	p < 0.0001	NS
Metabolic syndrome	p < 0.0001	/	ND	/	ND	/
Alcohol consumption	ND	ND	ND	Details not described	x2 = 7.15, p = 0.008	NS
Abnormal LFT	ND	ND	ND	Details not described	p < 0.0001	NS
Fatty liver	ND	ND	ND	Details not described	p < 0.0001	NS

Criteria to define healthy						

MS excluded		Yes		Yes		Yes
HF excluded		No description		Yes		Yes
Normal LFT		Yes		Yes		Yes
Alcohol consumption (g/d)		≤30		No		≤20
BMI (kg/m2)		non-obese(< 30)		No		< 23
Exclusion of fatty liver		No		Yes by CT scan		Yes by ultrasonography

Liver stiffness (mean ± SD)	5.81 ± 1.54 (male) 5.23 ± 1.59 (female)	5.60 ± 1.30 (male) 5.05 ± 1.49(female)	ND	4.5 ± 0.4	4.88 ± 1.77(male) 4.52 ± 1.11(female)	4.30 ± 0.81

Normal upper limit of liver stiffness		8.0 (male, 90 percentile) 7.8 (female, 90 percentile)		5.3(95th percentile)		5.9 (Mean + 2SD) 5.5 (Non drinker)

ND: not determined; NS: not significant; HF: heart failure; MS: metabolic syndrome

Kim et al. [17] established a normal liver stiffness value (4.5 ± 0.4 kPa) based on a study of 69 healthy liver and kidney donors who passed screening evaluations for transplantation, whose laboratory findings were normal, and who could be regarded as being healthy and free of liver disease. Our normal upper limits of liver stiffness were nearly equivalent to theirs, which was lower than that of Roulot et al. The study population and criteria for healthy controls shown in Table 4 probably can account for these differences.

Liver disorders are usually screened during routine medical checkups or in clinical practice using LF tests and ultrasonography. Figures 5, 6 and 7 show that LS (in addition to a standard set of LF tests and ultrasonography) can discriminate individuals at high risk for non-alcoholic and alcoholic liver diseases.

While investigations of the pathogenesis of various liver diseases are important, liver biopsies are not always feasible because of adverse effects, and were unobtainable from our population from an ethical standpoint. Nevertheless, our reference values for LS would be useful to select candidates for liver biopsy and thus decrease the rate of unnecessary procedures.

## Conclusion

In conclusion, our study uncovered several factors that are associated with LS, and showed that measuring LS is a non-invasive, simple and efficient method of identifying individuals at high risk for ALD and NAFLD. Future studies should investigate pathological factors other than fibrosis that influence LS in clinical populations.

## List of abbreviations

ALD: alcoholic liver disease; ALT: alanine aminotransferase; APRI: ratio of aspartate 2-oxoglutarate aminotransferase to platelet ratio index; AST: aspartate 2-oxoglutarate aminotransferase; BMI: body mass index; γ-GTP: gamma-glutamyltranspeptidase; HF: heart failure; LF: liver function; LS: liver stiffness; MS: metabolic syndrome; NAFLD: nonalcoholic fatty liver disease; NASH: nonalcoholic steatohepatitis.

## Competing interests

The authors declare that they have no competing interests.

## Authors' contributions

MB carried out this study, participated in the conception and design of the study. KF conceptualized, edited the manuscript for important intellectual content and has read and approved the final version of the manuscript. HB performed participated in the analysis and interpretation of the data. KK performed participated in the analysis and interpretation of the data. KS performed participated in the analysis and interpretation of the data. All authors read and approved the final manuscript.

## Pre-publication history

The pre-publication history for this paper can be accessed here:

http://www.biomedcentral.com/1471-230X/11/70/prepub
